# Automatic detection of false annotations via binary property clustering

**DOI:** 10.1186/1471-2105-6-46

**Published:** 2005-03-08

**Authors:** Noam Kaplan, Michal Linial

**Affiliations:** 1 Department of Biological Chemistry, Institute of Life Sciences, The Hebrew University of Jerusalem, Israel; 2 Department of Computer Science and Engineering, University of Washington, Seattle, WA, USA

## Abstract

**Background:**

Computational protein annotation methods occasionally introduce errors. False-positive (FP) errors are annotations that are mistakenly associated with a protein. Such false annotations introduce errors that may spread into databases through similarity with other proteins. Generally, methods used to minimize the chance for FPs result in decreased sensitivity or low throughput. We present a novel protein-clustering method that enables automatic separation of FP from true hits. The method quantifies the biological similarity between pairs of proteins by examining each protein's annotations, and then proceeds by clustering sets of proteins that received similar annotation into biological groups.

**Results:**

Using a test set of all PROSITE signatures that are marked as FPs, we show that the method successfully separates FPs in 69% of the 327 test cases supplied by PROSITE. Furthermore, we constructed an extensive random FP simulation test and show a high degree of success in detecting FP, indicating that the method is not specifically tuned for PROSITE and performs well on larger scales. We also suggest some means of predicting in which cases this approach would be successful.

**Conclusion:**

Automatic detection of FPs may greatly facilitate the manual validation process and increase annotation sensitivity. With the increasing number of automatic annotations, the tendency of biological properties to be clustered, once a biological similarity measure is introduced, may become exceedingly helpful in the development of such automatic methods.

## Background

Computational protein annotation is a major goal of bioinformatics and annotation methods are widely used. A wide variety of annotation methods exist, many of which rely on some kind of scoring. Typically, when testing whether a protein should be given a certain annotation, a score threshold is set, and proteins that score higher than the threshold are given the annotation. Obviously, some annotation mistakes may occur. Such mistakes can be divided into false positives (FPs) and false negatives (FNs). FPs (or false hits) are annotations that were mistakenly assigned to a protein (type I error). FNs (or misses) are annotations that should have been assigned to a protein but were not (type II error). Adjustment of score thresholds allows tradeoff between these two types of mistakes. FPs annotations are considered to be of graver consequence than FNs. This is partially due to the fact that introduction of a false positive annotation into a protein database may cause other proteins to become incorrectly annotated on the basis of sequence similarity [1,2]. A systematic evaluation of the source of false annotations that already contaminated current databases was reported [3]. Several automatic systems such as PEDANT [4] and GeneQuiz [5] were introduced with the goal of matching the performance of human experts. Still, over interpretation, FN errors, typographic mistakes and the domain-based transitivity pitfall [6] limit the use of such fully automatic systems for inferring protein function.

Due to the importance of minimizing the amount of false annotations and maintaining highly reliable protein databases, three methods are generally used to avoid false annotations. The first method is manual validation of the annotation of each protein, which creates a serious bottleneck in the addition of new proteins and annotations to the database. The second method is using high score thresholds, thus lowering the rate of FPs but also increasing the rate of FNs (resulting in a loss of sensitivity). The third method is requirement for hits from different detection methods, eliminating advantages that are unique to some methods. Thus it would be beneficial to develop means by which FP annotations could be detected automatically, allowing both high throughput and high sensitivity.

Here we present such a method that uses clustering of protein functional groups to separate true positives (TPs) from FPs automatically. Our method is based on the following notions: (a) protein annotations represent biological properties; (b) protein functional groups share specific combinations of biological properties, essentially constituting "property clusters"; (c) if two proteins have very different combinations of annotations, they are unlikely to share a single functional annotation and therefore there is a high chance that one of them was given that annotation incorrectly. These notions are not obvious, but were shown to correctly indicate false annotations in some individual cases tested manually using the graphical annotation-analysis tool of PANDORA [7]. We aim to generalize these sporadic observations and to address the feasibility of automating the detection of FP.

Using these ideas, the method attempts to separate a group of proteins into "property clusters", by introducing a measure that quantifies the similarity between the annotation combinations of two proteins. According to our basic notions, these clusters are likely to be in accordance with false and true hits.

We tested our method on the PROSITE protein signature database [8]. The database consists of 1189 protein signatures (essentially annotations) that were assigned to a protein database. PROSITE annotation of proteins is manually validated, stating for each protein hit whether the annotation is a TP or a FP. Out of this set of 1,189 signatures, we chose a subset of all signatures that have both true and false hits, and this served as our test set. Altogether 327 such signatures were collected and tested. For each of the signatures, the method examined the set of proteins that were assigned the signature. We called the separation successful only if at any step of the clustering process all the TPs were clustered together without any FPs. We applied a stringent scoring, where a partial success is considered failure.

Furthermore, we constructed a random FP simulation test in order to provide a more extensive test. In this test, all 5,551 InterPro [9] annotations were considered. For each InterPro annotation we selected the set of proteins in SwissProt [10] that were assigned that annotation, and added to that set random proteins, simulating proteins that were assigned the annotation by mistake (FPs). For each annotation we repeated the test 15 times: 5 times with 1 random protein, 5 times with 5 random proteins and 5 times with 10 random proteins. This artificial contamination of the annotation source strives to simulate mistaken annotations that may occur under some automation annotation inference schemes.

## Results

### Property-based clustering

We begin by describing the method of property-based clustering. Given a set P of all proteins that were given a certain annotation, and that there are both FPs and TPs in P, we would like to separate the set into disjoint subsets, so that one of the subsets will include all TPs and no FPs (leaving one or more subsets containing the FPs).

Annotation-based clustering is used to detect these subsets. We define an annotation as a binary property assigned to a protein (each protein may or may not have a "hit"). At the first stage, annotations from GO (Gene Ontology) [11], InterPro (entries) and SwissProt (keywords) are gathered for all proteins in P.

The clustering works in the following way: between each two proteins we define a similarity score that tries to quantify how much do the two proteins have in common from a biological perspective. The score between two proteins *p*_1 _and *p*_2 _is defined as:


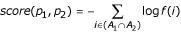


where *A*_1 _and *A*_2 _are the set of annotations of proteins *p*_1 _and *p*_2 _respectively, *i *is the current annotation, and *f*(*i*) is the frequency of *i *in the database. This score uses the following logic: if two proteins share an annotation, they are biologically similar in some manner. The more annotations these proteins share, the more cause we have to believe that they are similar biologically. However, two proteins sharing an annotation like "Enzyme" (that appears 45,991 times in our database) should receive a worse similarity score than two proteins that share a much uncommon annotation like "Heat Shock Protein" (that appears only 832 times). This is taken into account by using log(*f*(*i*)). Obviously, one could think of different scoring schemes that would quantify this differently. For a specific example of how the score is calculated see Table 1.

The similarity score is calculated between every two proteins in P. Next, we define the similarity score between two clusters as the arithmetic average of scores of all inter-cluster protein pairs:


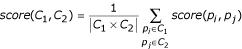


where *C*_1 _and *C*_2 _are clusters of proteins. Starting with clusters of 1 protein each, the method begins by an initial one-step clustering which merges all clusters that have the exact same combination of annotations. Following this the primary clustering commences: At each clustering step the two clusters that have the highest similarity score are merged. At each step the contents of the clusters are evaluated, and if all TP proteins appear in one cluster without any FPs, we say that the clustering process successfully separated the TPs from the FPs. Note that we do not require all the FPs to be grouped into one cluster, due to the fact that they cannot be expected to share biological similarity amongst themselves.

### PROSITE test

Out of 327 sets of proteins that share a PROSITE signature, the method showed successful separation (as defined previously) in 227 sets, i.e. 69% of the cases. The average size of the protein sets was 156.1 and the median 76. Altogether 58,254 proteins were used for this test. The average and median FP rates (FP rate is defined as: FP/(TP+FP)) of the sets were 0.12 and 0.07 respectively. These general statistics about the test set indicate that the sets were large enough and had a high enough amount of TPs and FPs so that the chance of random success would be minimal.

**Figure 1:**
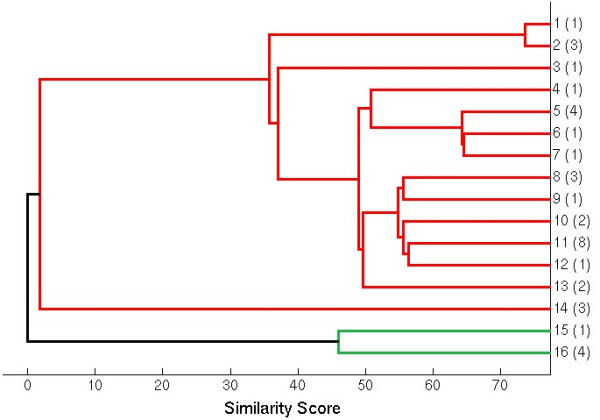
**Biological clustering example. **The figure shows a dendrogram describing the clustering of 37 proteins that matched the PROSITE "Serum Albumin Family" signature. The clustering advances from right to left along the axis that shows the similarity score at each point of the process. The vertical axis shows 16 initial clusters of proteins that the clustering starts with after the initial clustering stage. The initial clusters are numbered 1–16 and in parentheses show the number of proteins in them. Clusters 1–3 contain 5 Vitamin D Binding proteins (TPs). Clusters 4–13 contain 24 Albumin proteins (TPs). Cluster 14 contains 3 Afamin proteins (TPs). Clusters 15 and 16 contain the 5 FPs. The colors indicate the correct separation of this set into TPs and FPs.

In order to demonstrate the method's performance in this test, we provide the following example of testing a single protein set. The set presented here is the set of all 37 proteins that matched the PROSITE "Serum albumin family" signature. Each protein in the set contains an average of 18.2 annotations (obviously not all are relevant). First, the score between every pair of proteins is calculated, based on their mutual annotations. Next, the proteins undergo a preliminary clustering step in which all proteins that have the exact same combination of annotations are merged into clusters. Following this, the proteins are clustered together based on their mutual similarity score. Finally, once the clustering has finished we examine the tree to see if the true positives were separated from the false positives. In the given example, there are 5 proteins that were incorrectly assigned the PROSITE annotation (FPs), and in Figure 1 we see that they are indeed separated from the TP proteins.

### Random FP simulation test

5,551 sets of proteins were tested 15 times each and showed successful separation in 74% of the cases. Altogether 99,076 proteins were used for this test. This can be subdivided into 78% success for the sets that had 1 random protein added, 74% success for the sets that had 5 random proteins added and 68% for the sets that had 10 random proteins added. The average set size was 78 proteins. The drop in the performance by increasing the level of FPs is due to the fact that there is a higher chance that one of the randomly selected proteins will be biologically similar to the TPs. Since we consider only cases in which all FPs are detected, then there would be a higher chance of failure as the number of randomly-generated FPs increases.

While the simulation of FP errors randomly provides endless amounts of test sets, which is a clear advantage over the limited test sets provided by a real database such as PROSITE, the simulation has its own limitations. The hidden assumption made by this approach is that the FP hits are independent of each other. This assumption is not necessarily true: for example, if annotation is done by means of sequence similarity, false hits may be more likely to be biologically similar to each other (e.g. belong to the same family). In fact, in many cases in the PROSITE test we find that the correct separation separates the TP proteins into one cluster and the FP into one or two clusters, suggesting that the FPs share some degree of biological similarity (see "Determination of the correct halting step"). This difference in the way that FP annotations are generated may also account for the difference in success rates between the PROSITE test set and the simulated test set. The way FP annotations are introduced into databases is impossible to model, but the combined success of the method on both a real database test set and on an extensive simulated test set seems promising.

A further issue which concerns the simulation method is determining the amount of FPs to add to each set. Here we chose to add 1, 5 or 10 proteins to each set. This does not necessarily reflect the amount of FPs in real databases. Understandably, each database's average FP rate depends on its specific characteristics. However, the PROSITE database's average FP rate of 0.12 (median of 0.08) might give an indication as to what a typical rate is. In comparison, the average FP rate for our random simulation set was 0.11 (median of 0.07), which suggests that our choice was reasonable.

### Determination of the correct halting step

**Figure 2:**
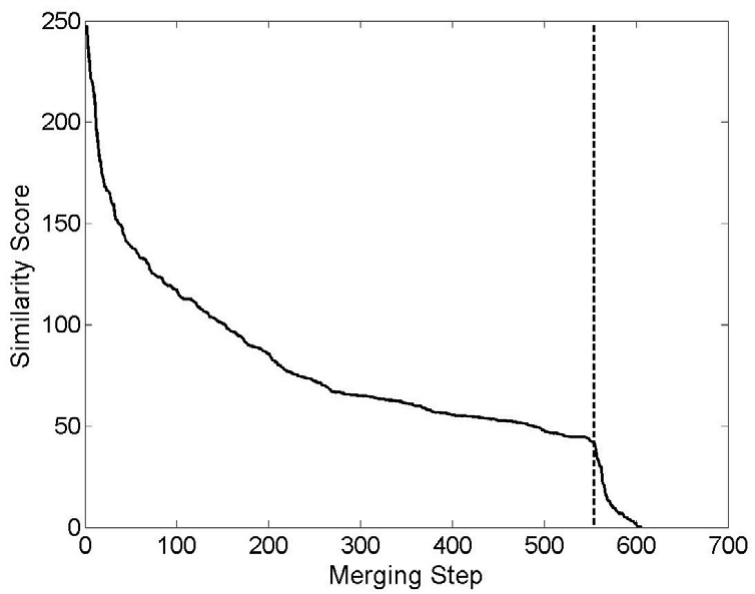
**Similarity score plot. **The figure shows the similarity score (solid line) plotted versus the progression of the clustering process for a sample protein set that was tested. The protein set includes 606 proteins that were annotated as "Rhodopsin-like GPCR superfamily". The score decreases from left to right as the clusters are merged, indicating decreasing biological similarity. The vertical dashed line indicates the correct halting step. Note that the correct halting step is located where there is a distinct knee in the graph, indicating a point of stability in the process.

We call a clustering process successful if it managed at any step to separate the false annotations. However, this step must be somehow determined automatically. There are two approaches to this: one is to use an intrinsic parameter of the clustering process that would indicate where the correct halting step is located; the other is selecting a predetermined step of the process. We chose the similarity score at each merging step as an intrinsic process parameter. When plotting the score against the progression of the clustering (Figure 2), a knee shape in the plot would indicate a point of stability (biological similarity), suggesting it as a potential halting step. Analysis of the second derivative of this plot allows finding these knee-shaped stability points automatically. Using this method, 56% percent of the correct halting steps in the PROSITE test were correctly predicted. A different approach was to always choose the last step or the last two steps as the correct halting step. This resulted in 45% and 65% correct prediction, respectively. Furthermore, the union of the correct predictions made by both approaches indicates that together they correctly predict the halting step in 79% of the PROSITE test cases.

## Discussion

### Prediction of success

Interestingly, we found that with certain sets the method tended to be more successful than with other sets, probably indicating that these sets are more coherent biologically. This might suggest exploring an approach in which for each annotation one could predict the level of success provided by this method. Furthermore, we used the InterPro categorization of annotations into types in order to check success in specific annotation types. InterPro divides its annotations into different categories, such as "domain", "repeat" and "family". Understandably, "family" type annotations had a ~30% higher success rate than the other annotation types, primarily due to the fact that the "family" annotations often represent protein sets that are biologically coherent whereas other types such as "repeat" or "domain" annotations are biologically diverse. This result would be expected by a method that performs a clustering based on biological similarity. This indicates that this approach should be aimed primarily at functional family annotations.

**Figure 3:**
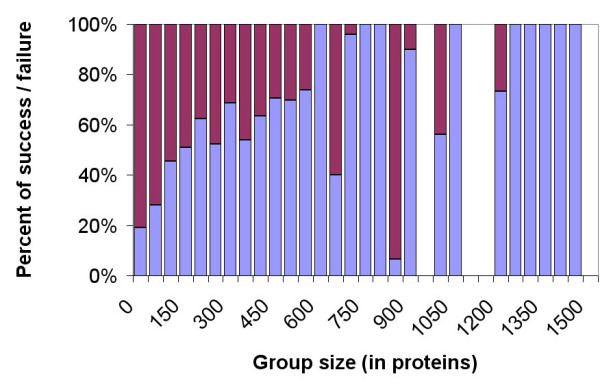
**Relative success and failure in group size categories. **The figure shows the relative success and failure of the clustering method in different categories of group sizes (the group size of an annotations is the number of proteins that have the annotation). All tested sets were grouped into 30 categories according to the amount of proteins in them, from 0 to 1500 proteins (shown on the horizontal axis). Each category shows the relative amount of success (purple) and failure (blue) of the method in each of the categories. It is apparent that relative success decreases as the group sizes increase.

However, functional families can be defined at different resolutions: an alcohol dehydrogenase belongs to the enzyme family, the dehydrogenase family and the alcohol dehydrogenase family. The test sets of the PROSITE and InterPro databases mainly represent mid-level and low-level annotations, with a typical size of tens or a few hundreds of proteins (see the statistics given previously). In order to further our understanding of the resolution in which this method is successful, we divided the protein groups into size categories and studied the relative amount of success in every size category. Figure 3 shows that as the group size increases, the rate of success decreases. Assuming larger sets represent the higher level annotations of InterPro, this suggests that when the annotations are more general ("higher" in the biological functional hierarchy) they have less in common biologically. Therefore, we would not expect the method to succeed on very general terms such as "enzyme". Sporadic tests of several high level GO annotations suggest that this is indeed the case (data not shown).

### Annotation source interdependency

Because multiple annotation sources were used, concerns arose regarding interdependencies amongst them. For example, InterPro is highly dependent on PROSITE, so proteins that have a PROSITE annotation will very likely be assigned an InterPro annotation as well automatically. In order to minimize this effect, we did not allow the algorithm to use the InterPro annotations that matched the PROSITE annotation which was being tested. Furthermore, in order to increase reliability of the random FP simulation test, all known PROSITE FPs were removed from InterPro prior to the test. Still, there is some concern that the results are partially biased due to annotation source interdependencies. Furthermore, it is difficult to determine whether these dependencies represent true biologically dependent properties, or simply a duplication of the same property in different sources. Keeping this difficulty in mind, our results which show different levels of success for different types of annotations (see "Prediction of success") indicate that the success of the method is more likely due to biological dependency rather than artificial duplication.

### Sufficient annotation

It should be stressed that the clustering process is based on sufficient annotation. Therefore, it may be difficult to apply this method to proteins that are poorly annotated. Still, these cases should be relatively rare: Nearly 77% of the ~1,600,000 proteins in TrEMBL [10] have at least one annotation by InterPro, and when considering several annotation sources there are on average ~10 annotations per SwissProt protein. Note that the amount and richness of annotation is constantly increasing at a fast rate. Furthermore, the ability to detect false annotations automatically may allow an increase in the sensitivity of current methods, thereby allowing more extensive annotation of proteins.

It is worthwhile noting that amongst the 58,254 proteins used in these sets there were 3,587 (6%) proteins annotated by SwissProt as "hypothetical proteins". 18% of the sets that were successfully separated contained such hypothetical proteins, with an average of 8% hypothetical proteins for each such set. These results suggest that the method is capable of handling to some extent hypothetical proteins of unknown function.

Another helpful approach to the problem of insufficient annotation could be the introduction of quantitative protein properties that are easily determined and show some correlation with function (i.e. the protein length, its Isoelectric point, etc.) into this method. Preliminary testing showed some positive correlation between protein length and Isoelectric point with function in certain cases (not shown).

## Conclusion

Introduction of FP annotations into protein databases can be harmful. It has been shown that once a mistaken annotation is introduced into a database, it often transfers to other proteins that are sequentially similar causing a propagation of false annotation [1]. Due to the importance of keeping high-quality databases, either the proteins are manually checked one by one or the annotation detection sensitivity is reduced in order to minimize FPs. The error rate and the limited sensitivity of assigning structural annotations using PSI-BLAST [12] or SAM-T98 [13] and methodologies based on HMMs and SVMs had been reported [14]. Naturally the process of manual validation of the annotation of protein databases is extremely time-consuming and in many cases is subjective to the expert view. Automatic detection of false annotations greatly facilitates the task of manual validation of annotation, and allows using lower thresholds when trying to detect protein signatures, therefore allowing higher method sensitivity.

Based on the notion that protein functional groups share specific combinations of annotations, we have introduced a method that by separating a set of proteins into biological "property clusters" shows successful separation of incorrectly annotated proteins from correctly annotated proteins. We test the method both with a manually validated test set and with a randomly constructed test set, and in both cases show a high degree of success. These results suggest that this tendency of certain annotations to appear in groups may be used as a basis of automatic methods that detect FPs. Naturally, different computer learning methods can be used to take advantage of these interdependencies of biological properties (for example see [15]).

## Methods

### Sources

We created a database that includes all proteins from SwissProt 40.28 (114,033 proteins) [10]. The database also included annotation of these proteins by GO[11], SwissProt and InterPro [9]. GO terms represent a wide range of biological terms concerning molecular function, cellular localization and biological processes, and span various degrees of specificity: from very general terms to very specific ones. GO terms are assigned to proteins both manually and automatically. InterPro annotations are assigned automatically by sequence and typically represent functional families and domains of no more than a few hundred protein members. SwissProt keywords are assigned manually and cover various biological subjects.

Annotation source and the number of annotation for each (in parenthesis) are: SwissProt version 40.28 (865 keywords), InterPro version 5.2 (5,551 entries), GO as of July 2002 (5,229 terms), PROSITE version 17.5 (1,189 signatures).

## Authors' contributions

NK and ML conceived of the study. NK designed the method. NK implemented and developed the method. NK designed the tests and analyzed the results. NK and ML wrote the manuscript.

## Figures and Tables

**Table 1:** Similarity score calculation

**Source**	**Annotation**	**CD63_RABIT^a^**	**CD68_HUMAN^a^**	**Frequency^b^**	**-ln(freq)**
**SwissProt**	Antigen	1	1	0.007130	4.9435114
**SwissProt**	Lysosome	1	1	0.001929	6.2506136
**SwissProt**	Glycoprotein	1	1	0.094727	2.3567562
**SwissProt**	Transmembrane	1	1	0.159770	1.8340200
**SwissProt**	Alternative splicing	0	1	0.029281	-
**SwissProt**	Signal	0	1	0.123850	-
**SwissProt**	Repeat	0	1	0.078968	-
**InterPro**	Serum albumin family	1	0	0.000342	-
**InterPro**	CD9/CD37/CD63 antigen	1	0	0.000666	-
**InterPro**	Lysosome-associated membrane glycoprotein (lamp)/CD68	0	1	0.000123	-
**GO**	Membrane	1	1	0.210869	1.5565182
**GO**	Lysosome	1	1	0.002043	6.1932038
**GO**	Vacuole	1	1	0.002184	6.1267895
**GO**	Lytic vacuole	1	1	0.002043	6.1932038
**GO**	Cell	1	1	0.440206	0.8205125
**GO**	Integral membrane protein	1	1	0.160874	1.8271338
**GO**	Cytoplasm	1	1	0.186569	1.6789541
**GO**	Intracellular	1	1	0.307578	1.1790266
				**Similarity Score:**	**40.960244**

The table shows a calculation of the similarity score between two SwissProt proteins: Rabbit CD63 antigen (CD63_RABIT) and Human Microsialin precursor (CD68_HUMAN). The similarity score is the summation of -ln(freq) on all annotations that are shared by both proteins. ^a ^– 1 or 0 indicate if the given protein has or does not have the annotation respectively. ^b ^– The frequency is the portion of proteins in the database that have the annotation.

## References

[B1] Linial M (2003). How incorrect annotations evolve-the case of short ORFs. Trends Biotechnol.

[B2] Gilks WR, Audit B, De Angelis D, Tsoka S, Ouzounis CA (2002). Modeling the percolation of annotation errors in a database of protein sequences. Bioinformatics.

[B3] Iliopoulos I, Tsoka S, Andrade MA, Enright AJ, Carroll M, Poullet P, Promponas V, Liakopoulos T, Palaios G, Pasquier C, Hamodrakas S, Tamames J, Yagnik AT, Tramontano A, Devos D, Blaschke C, Valencia A, Brett D, Martin D, Leroy C, Rigoutsos I, Sander C, Ouzounis CA (2003). Evaluation of annotation strategies using an entire genome sequence. Bioinformatics.

[B4] Frishman D, Mokrejs M, Kosykh D, Kastenmuller G, Kolesov G, Zubrzycki I, Gruber C, Geier B, Kaps A, Albermann K, Volz A, Wagner C, Fellenberg M, Heumann K, Mewes HW (2003). The PEDANT genome database. Nucleic Acids Res.

[B5] Andrade MA, Brown NP, Leroy C, Hoersch S, De Daruvar A, Reich C, Franchini A, Tamames J, Valencia A, Ouzounis C, Sander C. (1999). Automated genome sequence analysis and annotation. Bioinformatics.

[B6] Devos D, Valencia A (2001). Intrinsic errors in genome annotation. Trends Genet.

[B7] Kaplan N, Vaaknin A, Linial M (2003). PANDORA: keyword-based analysis of protein sets by integration of annotation sources. Nucleic Acids Res.

[B8] Sigrist CJ, Cerutti L, Hulo N, Gattiker A, Falquet L, Pagni M, Bairoch A, Bucher P (2002). PROSITE: a documented database using patterns and profiles as motif descriptors. Brief Bioinform.

[B9] Apweiler R, Attwood TK, Bairoch A, Bateman A, Birney E, Biswas M, Bucher P, Cerutti L, Corpet F, Croning MD, Durbin R, Falquet L, Fleischmann W, Gouzy J, Hermjakob H, Hulo N, Jonassen I, Kahn D, Kanapin A, Karavidopoulou Y, Lopez R, Marx B, Mulder NJ, Oinn TM, Pagni M, Servant F, Sigrist CJ, Zdobnov EM (2000). InterPro – an integrated documentation resource for protein families, domains and functional sites. Bioinformatics.

[B10] Boeckmann B, Bairoch A, Apweiler R, Blatter MC, Estreicher A, Gasteiger E, Martin MJ, Michoud K, O'Donovan C, Phan I, Pilbout S, Schneider M (2003). The SWISS-PROT protein knowledgebase and its supplement TrEMBL in 2003. Nucleic Acids Res.

[B11] Camon E, Magrane M, Barrell D, Binns D, Fleischmann W, Kersey P, Mulder N, Oinn T, Maslen J, Cox A, Apweiler R (2003). The Gene Ontology Annotation (GOA) Project: Implementation of GO in SWISS-PROT, TrEMBL, and InterPro. Genome Res.

[B12] Muller A, MacCallum RM, Sternberg MJ (1999). Benchmarking PSI-BLAST in genome annotation. J Mol Biol.

[B13] Karplus K, Barrett C, Hughey R (1998). Hidden Markov models for detecting remote protein homologies. Bioinformatics.

[B14] Karchin R, Karplus K, Haussler D (2002). Classifying G-protein coupled receptors with support vector machines. Bioinformatics.

[B15] Wieser D, Kretschmann E, Apweiler R (2004). Filtering erroneous protein annotation. Bioinformatics.

